# A simulator for spatially extended kappa models

**DOI:** 10.1093/bioinformatics/btt523

**Published:** 2013-09-09

**Authors:** Oksana Sorokina, Anatoly Sorokin, J. Douglas Armstrong, Vincent Danos

**Affiliations:** ^1^School of Informatics, University of Edinburgh, Edinburgh, UK and ^2^Institute of Cell Biophysics, Russian Academy of Sciences, Pushchino 142290, Russia

## Abstract

**Summary:** Spatial Kappa is a simulator of models written in a variant of the rule-based stochastic modelling language Kappa, with spatial extensions.

**Availability:** The spatial kappa simulator is an open-source project licensed under the LGPLv3, with Java source, binaries and manual available at http://github.com/lptolik/SpatialKappa.

**Contact:** oksana.sorokina@ed.ac.uk

**Supplementary information:** Supplementary data are available at *Bioinformatics* online.

The Kappa language is one of a family of rule-based modelling techniques that support mathematical models in the form of a collection of rules, where each rule represents only a salient subset of information about interactions of model species and modifications of their states. Thus, Kappa is well suited for describing highly combinatorial networks [e.g. ErbB signalling (Danos *et al.*, 2008)]. Additional tools (Danos *et al.,* 2009) allow the construction of models from descriptions of generic elements and rules (e.g. receptor and/or ligand families and interactions between them). Large and dynamic multimolecule protein complexes can be modelled in a precise and scalable manner (Sorokina *et al.,* 2011).

Spatial Kappa (SK) is an extension of Kappa, which supports spatial concepts in the form of voxel-based compartments, connections between them (known as channels) and translocation rules for species. This includes the motion of complex species spanning multiple voxels and compartments. Using these extensions, models can be constructed, which capture the importance of location and movement of species.

To support diffusion and transport, SK adds ‘next-subvolume’ diffusion (Elf *et al.,* 2003) to the Kappa framework. This positions SK among other meso-scale modelling techniques, e.g. MesoRD (Fange *et al.,* 2010) and SmartCell (Ander *et al.,* 2004). Several existing approaches handle spatially constrained models (Burrage *et al.,* 2011), but none builds on a rule-based approach, with the notable exception of cBNG which extends the BNG rule-based language (Harris *et al.,* 2009). However, unlike cBNG, SK does not assume that the contents of an individual compartment are well stirred; agents and complexes can be distributed heterogeneously.

We review the main language concepts (1–4) embodied in the simulator (5).

## 1 COMPARTMENTS AND VOXELS

**Fig. 1.**
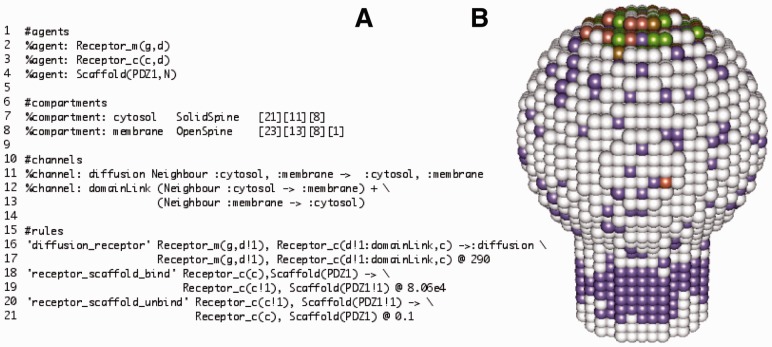
A snippet of an SK model of lateral diffusion of AMPA receptors on the postsynaptic membrane (**A**) Transmembrane receptors diffuse from the spine neck and bind scaffold proteins in the post-synaptic density, on the top of the cytosol compartment. Channels are used for cross-compartmental linkage (lines 12, 16) and multicompartmental diffusion (lines 11, 16). Snapshot taken after 2’ of simulation (**B**)

Compartments can be specified as single voxels (unit volumes) or as regular lattices of voxels. Once defined, compartments and voxels within a compartment can be referenced by rules. Most commonly used compartment shapes are predefined in 2D and 3D with both solid and hollow (open) modes. Examples include rectangles, circles, cuboids, spheres, cylinders, as well as custom shapes, e.g. dendritic spines (Fig. 1A, lines 7–8). The result is a lattice approximation of the specified shape ready for simulation. For open compartment types, thickness (Fig. 1A, line 8) should also be specified to constrain the reaction volume.

## 2 CHANNELS AND PATHS

Voxels within and across compartments can be linked to each other via channels. Channels describe both static links between agents in neighbouring voxels and movement of agents along predefined routes during active or diffusive transport (Fig. 1A, lines 11–12). A channel definition, thus, connects a set of source locations to a set of target locations. Multiple source voxels can be assigned to the same channel simultaneously if needed. For example, to represent the lateral movement of a transmembrane protein complex along the surface of a membrane, the channel would include coordinated displacement spanning two compartments: cytosol and membrane.

Commonly used intra-compartment channel types are predefined. These include nearest neighbours in 2D lattices (both Cartesian and hexagonal), nearest neighbours in 3D and directed nearest neighbours for radial or lateral diffusion within a compartment.

The predefined channel types can also be used for inter-compartment movement, subject to them having compatible geometries, free of overlapping voxel and gaps (Fig. 1A, line 12).

## 3 AGENT LOCATIONS, LINKS AND MOVEMENT

Species definitions from the original Kappa syntax can be partly or completely located within a defined geometry, at the onset of the simulation. Depending on the requirements, species can also be constrained to a single voxel or allowed to diffuse freely.

Agents can move along the defined channels. Translocation rules can be defined using species, locations or both. Diffusion of complexes that span several voxels is also supported. Agents in neighbouring voxels can be linked together via channels (Fig. 1A, line 15 ‘domainLink’). These spatial links can be created or broken dynamically like other Kappa links via transition rules. Species with cross-compartmental or cross-voxel links can diffuse along channels with multiple sources (Fig. 1A, line 11). In addition, the rate of a rule may contain an agent description. This allows one, for instance, to modulate the rate of a diffusion rule as a function of the size of the species being diffused.

## 4 SIMULATOR

The simulator underpinning SK was developed to provide the functionality described while maintaining compatibility with the reference Kappa simulator KaSim v3 (Feret and Krivine, 2013). Thanks to this, one can develop a space-less version of a model (which is natural when exploiting some form of proteomic data), and, in a second step, embed the model by building *ad hoc* geometries, channels and translocation rules. The SK simulator uses a variant of the next subvolume method (Ander *et al.,* 2004; Elf *et al.,* 2003), modifying the standard Gillespie stochastic simulation algorithm (Gibson and Bruck, 2000; Gillespie, 1977) to handle both reaction and diffusion transitions (see Manual for more details). The application outputs simulation results using a file format similar to KaSim, and also provides a simple time-series plot view updated during a running simulation. Running the simple model in Figure 1 (see also https://github.com/lptolik/SPKappaR/wiki/SuplementaryImages) can take from 30” to 10’ depending on the model parameters.

## 5 CONCLUSION

We have presented in this note SK, a language and simulator to embed rule-based models in space. Although equivalent features exist in other simulation tools, the complete feature set below is currently unique:

− Kappa-like rule-based model definition.
− Rule-based simulation for models with combinatorial complex constructions.
− Flexible compartment definitions support arbitrary dimensions and connectivity between compartments.
− Use of channels to simplify model construction: defined once, they can be used repeatedly within rules which involve a spatial element.
− Complex composition-based diffusion rates that can vary with size and composition of a complex to represent, e.g. complexes diffusing more slowly as they grow larger.
− Multicompartment complexes allowing for large aggregates or transmembrane arrangements.
− Diffusion of multicompartment complexes via special multisource channels.
− The tool for the 3D/4D visualization of SK models (see Fig. 1B) is available at https://github.com/lptolik/SPKappaR.

The formal description of the SK language, examples and the grammar can be found in the user manual distributed with the source code.

## Supplementary Material

Supplementary Data
