# Supplementary material for: A simulator for spatially extended kappa models
Source: Bioinformatics. 2013 Sep 9;29(23):3105–6. doi: 10.1093/bioinformatics/btt523 (PMC3834793; doi:10.1093/bioinformatics/btt523)
Supplement: Supplementary Data [file supp_29_23_3105__index.html]

A simulator for Spatially Extended Kappa Models — A simulator for spatially extended kappa models — A simulator for spatially extended kappa models — Supplementary Data 

# A simulator for spatially extended kappa models

## Supplementary Data

files

**Files in this Data Supplement:**

- Supplementary Data - pdf file
