# Supplementary material for: A simulator for spatially extended kappa models
Source: Bioinformatics. 2013 Sep 9;29(23):3105–6. doi: 10.1093/bioinformatics/btt523 (PMC3834793; doi:10.1093/bioinformatics/btt523)
Supplement: Supplementary Data [file supp_btt523_SpatialKappaManual.pdf]

Spatial Kappa Simulator[1]
User Guide
v2.1.1

Anatoly Sorokin[2] Vincent Danos[2] Oksana Sorokina[2]
Donal Stewart[3]

August 30, 2013

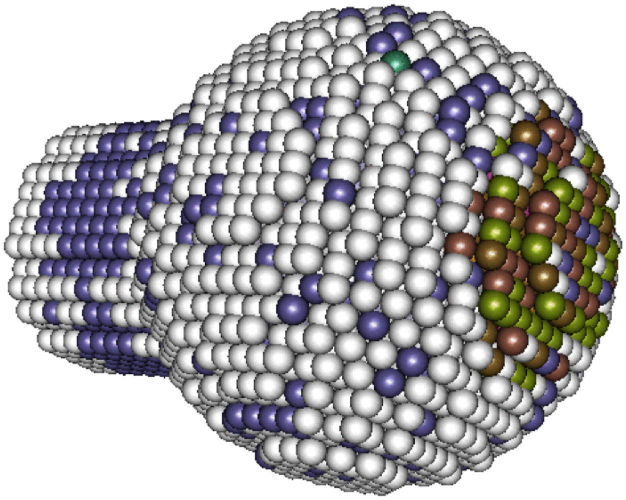

[1]The simulator is available from GitHub as the source Eclipse project, or as a single executable jar file. Both can be found at https://github.com/lptolik/SpatialKappa/.

[2]The University of Edinburgh, School of Informatics, Edinburgh, UK
[3]DemonSoft.org, UK

Acknowledgements

The development of Spatial Kappa was funded in part by the Edinburgh Centre for Synthetic and Systems Biology (SynthSys). SynthSys was funded as a Centre for Integrative Systems Biology (CISB) jointly funded by BBSRC and EPSRC (reference BB/D019621/1) for the period 2007–2012.

Contents

1 Kappa Language Extensions **4**
- 1.1 Existing Kappa language . . . 4
- 1.2 New Concepts . . . 5
- 1.3 New language constructs . . . 6
  - 1.3.1 Compartments and voxels . . . 6
  - 1.3.2 Channels . . . 9
  - 1.3.3 Locating agents . . . 12
  - 1.3.4 Agent links . . . 12
  - 1.3.5 Species movement . . . 13
  - 1.3.6 Instance specific reaction rates . . . 14
  - 1.3.7 Voxel breakdown of observables . . . 15

2 Spatial Kappa simulator User Guide **16**
- 2.1 Obtaining the simulator . . . 16
- 2.2 Starting the simulator . . . 16
  - 2.2.1 Running the executable jar . . . 16
  - 2.2.2 Running from the Eclipse project . . . 16
- 2.3 Using the simulator . . . 16
  - 2.3.1 Opening a Kappa or Kappa replay file . . . 17
  - 2.3.2 Running a simulation . . . 17
  - 2.3.3 Running a simulation replay . . . 18
- 2.4 Time series chart . . . 18

3 Spatial Kappa reference implementation **20**
- 3.1 Initialization . . . 20
- 3.2 Main simulation loop . . . 21
  - 3.2.1 Finite rate rules . . . 21

A Spatial Kappa Grammar **22**

B Spatial Kappa Examples **27**
- B.1 Spatial Kappa patterns . . . 27
  - B.1.1 1 dimensional patterns . . . 27
  - B.1.2 2 dimensional surfaces . . . 27
- B.2 Sample Spatial Kappa models . . . 30
  - B.2.1 2D diffusion model . . . 30
  - B.2.2 Bi-trivalent binding model . . . 30

What is rule-based modeling?

Rule-based modeling uses rules to construct models of systems of interacting bio-molecules. The approach is especially effective for systems with combinatorial complexity, where relatively concise rule-sets may approximate information about a complex biochemical network with enough precision. Proteins that participate in signaling cascades are commonly composed of multiple subunits and binding domains. These domains can mediate more than one domain-domain interaction, and/or allow for the formation or repeated polymeric motifs, which results in many possible protein complexes. Domain-domain interactions, in turn, are governed by post-translational modifications that provide an additional level of variety. Therefore, when conventional modelling, such as ODEs, takes these elements of molecular structure into account - model sizes grow exponentially (or worse) with the number of interactions. Instead, graphs can be used to conveniently represent structured objects (such as molecules with there sites and states) and graph-rewriting rules can approximate their interactions. A graph-rewriting rule specifies an edge addition or removal when proteins are binding or unbinding. It can also change the state (label) of any component to represent post-translational modification. Each rule accounts for the class of reactions that chemicals with common structure could share. Only information that is important for a particular interaction is taken into account, so that the states and domains that are not involved are ignored. All the reactions that are implied by one rule will be parameterized by the same rate constant, which gives a significant reduction of the model complexity.

What is Kappa?

Kappa is a member of the rule-based language family. Its main simulator is KaSim (Feret and Krivine, 2012) now in version 3. To know how plain Kappa is used in a modeling context, the following short note is a good start (Danos, 2009). A longer article is also available (Danos et al., 2007a); see also this tutorial (Krivine et al., 2009).

What is this User Guide about?

This manual documents an extension of the Kappa language, called *Spatial Kappa*. Differently from Kappa which assumes that all agents in a model co-exist in an homogeneous and well-stirred volume, in Spatial Kappa, one can define compartments and rules for diffusion within and transport across them.

The spatial Kappa language is backward compatible with basic Kappa. Potential users, already familiar with Kappa, should find the extension intuitive.

Existing Kappa models can be run without modifications —provided they do not incorporate directives related to causality analysis and causal flows. This allows one to develop a model in a space-less manner first, and to then introduce spatial aspects gradually.

Chapter 1

Kappa Language Extensions

Note on terminology: in the following 'voxel' means a subunit of a defined compartment; 'species' is used to refer either to a particular agent, or a partial complex of agents, or a full complex —when we need to be more explicit, we say agent, partial complex, or full complex; usual Kappa rules are sometimes called transform rules, as they entail the modification of their targets, to stress the difference with transport or diffusion rules which merely displace their targets.

1.1 Existing Kappa language

We start with a brief description of the current Kappa language as defined in the KaSim Reference Manual (Feret and Krivine, 2012). A formal description of the language including spatial extensions is given in Appendix A.

Basic elements in a model can be declared either as agents or tokens. *Agents* are used to represent complex objects with multiple binding sites that can bind/unbind and modify others. They have a discrete number of instances. *Tokens* correspond to simple unstructured objects. They cannot bind to other tokens and agents, and can only appear and disappear. They have continuous concentrations. An agent declaration contains its *signature*, that is to say the information about the agent that will be used in the model, such as its name, its interactions sites and their states. For instance, the line:

```
%agent: A (x,y~u~p,z~0~1~2)
```

declares an agent `A` with three binding sites `x`, `y` and `z`, where `y` can exist in two states (`u` and `p`, typically for phosphorylated and unphosphorylated) and `z` - in three states, `0`, `1`, and `2`. It is possible to declare an agent with any number of sites, including no sites at all (in which case the agent essentially a token).

Declared agents are used in turn to define rules which describe their dynamics. A typical rule starts with a name (a string) and contains a left hand side and a right hand side expression together with a corresponding kinetic rate.

```
'my rule' kappa_expression -> kappa_expression @ rate
```

Rules can also be bi-directional, in which case one must provide two kinetic rates:

```
'my bidirectional rule' kappa_expression <-> kappa_expression @ rate_left_to_right, rate_right_to_left
```

Rates can be (positive) real numbers as well as algebraic expressions. Here are examples of the most common types of rules:

```
'state change' A(state~old) -> A(state~new) @ 0.1
'binding'      A(bindsite),B(bindsite) -> A(bindsite!1),B(bindsite!1) @ 0.1
'unbinding'    A(bindsite!1),B(bindsite!1) -> A(bindsite),B(bindsite) @ 0.1
'creation'     -> A() @ 0.1
'degradation'  A() -> @ 0.1
```

The initial string, i.e. the name of the rule, is optional:

```
A(state~old) -> A(state~new) @ 0.1 # Unnamed rule
```

(Note above the use of a comment with comment character `#`.)

Initial conditions are defined in the form of algebraic expressions to be evaluated before the initialization of the simulation:

```
%init 1000 A(state~old),C()            # 1000 of each of A(..) and C()
%init 2000 A(bindsite!1),B(bindsite!1) # 2000 of the dimer A(..),B(..)
%var  'n' 100
%init 'n' (A(),A(y~p)) # 100 instances of A default state A (x,y~u,z~0) and 100 A(x,y~p,z~0)
```

The above snippet of code introduces a *variable* `'n'`. Once declared, a variable can be referenced in rates, observables, as well as in perturbations (see below). *Observables* define the outputs of the simulation.

```
%obs 'Label' A()  # All agents A()
%obs A()          # Unnamed observation, will default to 'A()' in outputs
%obs A(state~old) # All agents matching A(state~old)
%obs 'binding'    # Activity of the transform rule named 'binding'

%var 'Named variable' B()
```

Variables can be used to trigger specific events named *perturbations* during the simulation.

```
# When observable 'mRNA' drops below 50 set the rate of 'transcribe' to 0.5
%mod: 'mRNA' < 50 do 'transcribe' := 0.5
```

Perturbations can also condition events on the global time of the simulation:

```
# At time 5, change the rate of 'unbinding' to 10.0
%mod: [T] > 5 do 'unbinding':= 10.0
```

1.2 New Concepts

Spatial Kappa introduces a number of new features: compartments, voxels, and channels which are used to defined transport between voxels and compartments, as well as new kinds of 'transport' rules which exploit these additional features. We go briefly over an informal presentation of these spatial features before describing the syntax of the language in the next Subsection.

Compartments: A model may have multiple compartments each containing reacting species. Each compartment has a dimension and is subdivided into voxels. Dimensions range from 0-dimensional single voxels, to 1-, 2-, or 3-dimensional structures. Compartments with differences in size can be specified simply by assigning them different number of voxels, e.g. the reacting volume of a nucleus relative to the surrounding cytosol. Differences in shape can also be specified, for example the thin layer of cytosol next to the inner surface of the plasma membrane versus the rest of the cytosol. To create concisely more accurate models, predefined compartment shapes are available: circles, spheres and cylinders (hollow or filled), etc.

Channels: To establish both intra- and inter-compartment connections, the language incorporates a notion of channel. Intra-compartment channels allow the description of multiple

structures: 1-dimensional linear arrays or circles, 2-dimensional square or hexagonal meshes, cylinders or tori, 3-dimensional cubes, filled cylinders, spheres, etc. Commonly used inter- and intra-compartment channels can be named for easier reference.

Locating species: Species can be placed within compartments. For instance: a DNA complex can be made to reside within the nucleus; cell receptors can be limited to the plasma membrane. It is important to note that multi-agent species do not need not be confined to a single voxel or even a single compartment, but may span multiple neighbouring voxels. We refer to them as multi-voxel species.

Locating transform rules: Similarly, rules can be placed within compartments. For instance: transcription-related rules can be confined to the nucleus. The language also allows the same transform rule to be specified with different rates depending on the location of the reacting species (which can be useful for instance when some locations are more crowded).

Translocation rules: One can define rules for the (active or diffusive) transport and diffusion of species, including multi-voxel ones. These rules can be within or between compartments and must use previously described channels. To transport multi-voxel species, one needs channels with multiple voxels as sources. When a rule specifies the translocation of a list of $n > 0$ partial species F_i, $0 \leq i < n$, via a channel c with n input voxels, translocation will only happen if the full complexes F'_i, which F_i belongs to, are contained in the input voxels of c, If it is not the case, one of the F_is exceeds the inputs of c, this is a *clash* or null event, meaning the event is cancelled (and time advances). If it is thecase, the F'_i full complexes will move to their destination voxels. One cannot drag along agents not in the inputs of c, or let them lag behind, neither can translocation break links from the F'_is to such agents. It is the *"no drag, no lag, no break"* convention. Note that it is possible that $F'_i = F'_j$ if there are links across the corresponding voxels (links which might or might not belong to the F_is themselves). Note also that the translocation of the F'_is might lead to a situation where cross-voxels links are formed which have not been declared. This is another source of potential clashes and event cancellations. Rates can be made to depend on the translocated species size and composition.

Granularity of locations: It is possible to specify locations at the level of entire compartments or single voxels within a compartment. This allows models to represent, for example, a signalling cascade being initiated at one point in the cytosol, and the resulting signal molecules being diffused through the cytosol.

1.3 New language constructs

A full BNF description of the extended Kappa grammar is given in Appendix A. The new constructs are identifiable as new types of statement (`%channel` and `%compartment`) and rules, and location or channel identifiers in existing rule types prefixed with '`:`'.

Warning - the Spatial Kappa parser expects a file to end with an empty line.

1.3.1 Compartments and voxels

Compartments are defined as single voxels or regular multidimensional arrays of voxels as follows

```
'%compartment:' name=id ('[' INT ']')*
```

For example:

```
%compartment: SingleCell
%compartment: line  [10]       # 10 voxels in size
%compartment: plane [10][5]    # 10x5 voxels in size
%compartment: box   [10][5][4] # 10x5x4 voxels in size
```

Compartments or individual voxels within a compartment can be referenced using the following location syntax (the reader not familiar with formal grammars can jump directly to the examples):

```
':' id ( '[' cellIndexExpr ']' )*
```

where

```
cellIndexExpr :
  cellIndexAtom operator_cell_index cellIndexAtom | cellIndexAtom

cellIndexAtom :
  '(' cellIndexExpr ')' | INT | id

operator_cell_index :
  '+' | '-' | '*' | '/' | '%'
```

For example:

```
:myCompartment                     # the compartment as a whole
:myCompartment [0][0][0]           # the first voxel in a 3D array compartment
:myCompartment [4]                 # not a legal location, see below

:myCompartment [x][y][z]           # variable name usage described in
:myCompartment [x*2][y -1][z +(x*3)] # channel section below
```

When locations are used, it is only legal to refer to the compartment as a whole by omitting the cell indices, or to refer to a single voxel by proving as many cell indices as the dimension of the compartment. Thus the third line above is illegal. Also, variable names are only permitted in locations within channel definitions, described below.

Warning - in the example above, you can see that there are spaces before the `+` and `-` operators, e.g. in `y -1`; this space is necessary, as the parser reads `y-1` as the name of a variable.

Predefined compartment types

Commonly used non-rectangular compartments can also be concisely defined. These include both solid and hollow (open) shapes in 2 and 3 dimensions. The available predefined compartment types are described in Table 1.1.

This allows the creation of a voxellated approximation of the shape specified, which can then be used for simulation. For the open compartment types, thickness specifies how thick in voxels the compartment reaction volume is.

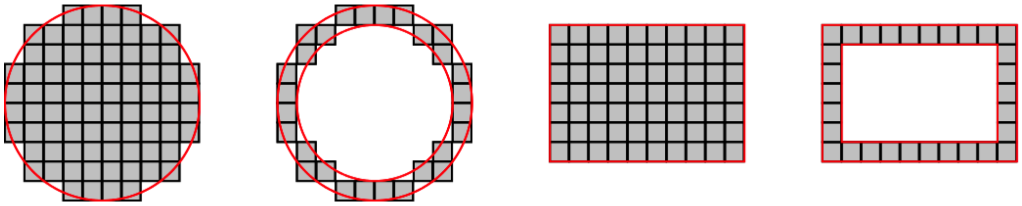

Figure 1.1: 2D compartment types (open and solid)

Table 1.1: Predefined compartments and their parameters.

Name	Parameters			
2D				
OpenRectangle	height	width	thickness	
SolidCircle	diameter			
OpenCircle	diameter	thickness		
3D				
OpenCuboid	height	width	depth	thickness
SolidSphere	diameter			
OpenSphere	diameter	thickness		
SolidCylinder	diameter	length		
OpenCylinder	diameter	length	thickness	

The syntax to specify predefined compartments is the same as above, except that one needs to specify the type of compartment and provide the necessary arguments.

```
'%compartment:' name=id type=id ('[' INT ']')*
```

This is best seen with examples:

```
### 2D Shapes

%compartment: solidRectangle                [10][5]        # [height][width]
%compartment: openRectangle OpenRectangle [10][5] [2]    # [height][width] [thickness]
%compartment: solidCircle   SolidCircle   [10]           # [diameter]
%compartment: openCircle    OpenCircle    [10] [2]       # [diameter] [thickness]

### 3D Shapes

%compartment: solidCuboid                 [10][5][8]     # [height][width][depth]
%compartment: openCuboid    OpenCuboid    [10][5][8] [2] # [height][width][depth] [thickness]
%compartment: solidSphere   SolidSphere   [10]           # [diameter]
%compartment: openSphere    OpenSphere    [10] [2]       # [diameter] [thickness]
%compartment: solidCylinder SolidCylinder [10][8]        # [diameter][length]
%compartment: openCylinder  OpenCylinder  [10][8] [2]    # [diameter][length] [thickness]
```

1.3.2 Channels

The structure of a compartment is complemented by the definition of *channels* which define how voxels are linked within and across compartments. Channels are used in defining both static links between agents in different voxels, and the motion of agents through the geometry of the model. The syntax is as follows:

```
'%channel:' id channel
| '%channel:' id '(' channel ')' ('+' '(' channel ')')*
```

where

```
channel :
  source=locations '->' target=locations

locations :
  location (',' location)*
```

Where `location` is as described above. For example:

```
%compartment: torus [10][200]    # 10x200 voxels in size

# Link all voxels to their horizontally adjacent neighbours
# Link all voxels to their vertically adjacent neighbours
# Wrap around the voxels on the left and right edges to create a cylinder
# Wrap around the voxels on the top and bottom edges to create a torus
%channel: meshlinks \
    (:torus[x][y] -> :torus[x +1][y]) + (:torus[x][y]   -> :torus[x -1][y]) + \
    (:torus[x][y] -> :torus[x][y +1]) + (:torus[x][y]   -> :torus[x][y -1]) + \
    (:torus[0][y] -> :torus[9][y])    + (:torus[9][y]   -> :torus[0][y])    + \
    (:torus[x][0] -> :torus[x][199])  + (:torus[x][199] -> :torus[x][0])
```

The above code defines a thin torus composed of a 2D mesh. (Note the use of \ to extend a command beyond a line.)

Locations on the left hand side (lhs) of the channel definitions above may contain either constant values or single variable names; complex expressions are forbidden and repeated variables are ignored and treated as new ones. The variable names are used to define the dimensions which will be iterated through to produce links. Locations on the right hand side (rhs) allow constant values or complex expressions involving the variables defined on the left hand side of the expression. It is invalid for the rhs to use variables not defined in the lhs. If setting the values of variables references valid voxels on both the left and right, then those voxels are deemed to be

linked. References to voxels *outside* the dimensions of the compartment are ignored, and no link is created. Locations referenced in a channel definition can belong to the same compartment, thus defining an intra-compartment channel, or to different compartments. The modulus operator `%` is useful in defining repeated patterns of linkage within a compartment, for example the 2D hexagonal mesh described in Appendix B.1.

Channels can make use of multiple source voxels simultaneously. For example if a model was to represent the movement of transmembrane proteins laterally along the surface of a membrane, then the channel used to describe the lateral motion would need to include simultaneous movement in two compartments (cytosol and membrane). This is represented as follows:

```
%compartment: membrane [5][5]
%compartment: cytosol  [5][5][5]

%channel: diffusion \
    (:membrane [x][y], :cytosol [u][v][0] -> :membrane [x +1][y], :cytosol [u +1][v][0]) + \
    (:membrane [x][y], :cytosol [u][v][0] -> :membrane [x -1][y], :cytosol [u -1][v][0]) + \
    (:membrane [x][y], :cytosol [u][v][0] -> :membrane [x][y +1], :cytosol [u][v +1][0]) + \
    (:membrane [x][y], :cytosol [u][v][0] -> :membrane [x][y -1], :cytosol [u][v -1][0])
```

Here, variables `x`, `y` represent locations in the membrane, and `u`, `v` represent locations in the top layer of the cytosol. The definition updates the locations in these two compartments in unison, resulting in the declaration of 5^4 different 2-input channels. Note that there must be the same number of voxels in the source and target of a channel (two in this example), and thus, channels define a one-one mapping between source and target voxels.

Further examples of compartment and channel specifications for common structures are given in Appendix B.1.

Predefined channel types

Commonly used 2- and 3-dimensional channel types can be concisely defined.

Table 1.2: Predefined channels.

Name	Each voxel connected to
2D	
EdgeNeighbour	4 neighbours which share an edge in grid
Hexagonal	6 neighbours which share an edge in hexagonal grid
Neighbour	8 neighbours which share an edge or corner in grid
3D	
FaceNeighbour	6 neighbours which share a face in grid
Neighbour	26 neighbours which share a face, edge or corner in grid

There are also predefined directional channel types usable in both 2D and 3D compartments

Name	Each voxel connected to
Radial	neighbours both directly towards and away from compartment centre
RadialIn	neighbours both directly towards compartment centre
RadialOut	neighbours both directly away from compartment centre
Lateral	neighbours at same distance from compartment centre

The syntax to use a predefined channel type is

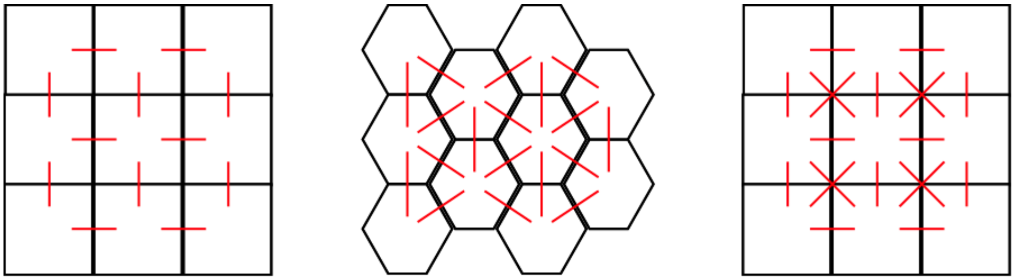

Figure 1.2: 2D channel types: EdgeNeighbour, Hexagonal and Neighbour

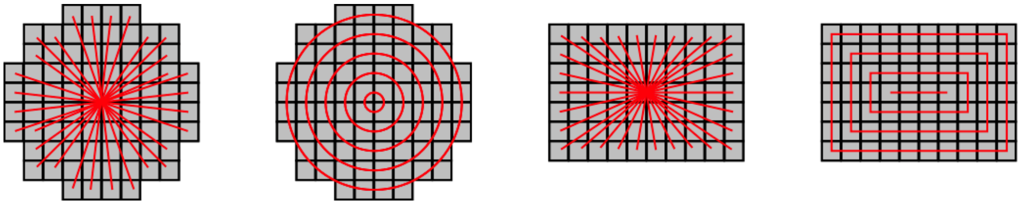

Figure 1.3: Directed channel types: Radial and Lateral

```
'%channel:' id channel
| '%channel:' id '(' channel ')' ('+' '(' channel ')')*
```

where

```
channel :
  type=id source=locations '->' target=locations
```

Where `locations` is as described above. For example:

```
%compartment: solidRectangle[5][5] # [height][width]

%channel: radial Radial :solidRectangle -> :solidRectangle
%channel: radialIn RadialIn :solidRectangle -> :solidRectangle
%channel: radialOut RadialOut :solidRectangle -> :solidRectangle
%channel: lateral Lateral :solidRectangle -> :solidRectangle
```

Using predefined channel types between compartments

It is possible to use predefined channel types (usually 'Neighbour' see also Table 1.2) to describe movement between compartments. For this to be valid, the compartments must have compatible geometry. Compatible compartments are treated as being nested one inside the other and the channel then specifies the diffusion (or agent linkage) across the boundary between the two compartments. The compartments must fit together exactly, with no overlapping voxels or gaps. For example, a circle may only be nested inside a larger open circle whose dimensions (diameter and thickness) allow the smaller circle to fit exactly inside.

```
%compartment: cytosol   SolidCircle   [3]        # [diameter]
%compartment: membrane  OpenCircle    [7] [2]    # [diameter] [thickness]

%channel: domainLink Neighbour (:cytosol -> :membrane) + (:membrane -> :cytosol)
```

1.3.3 Locating agents

The definitions above can now be used to locate species within the model. Any rule or directive (i.e., variables, observables, and initial conditions) that accepts agents as part of its definition, now allows these agents to be located. For each group of agents, a prefixed location constrains the agents to that location. For example:

```
%compartment: membrane [5][5]
%compartment: cytosol  [5][5][5]

%init: 1000 A                    # A distributed evenly among all voxels in model
%init: 1000 :cytosol B           # B distributed evenly among all voxels in cytosol
%init: 1000 :membrane[2][2] C    # C in one voxel of the membrane only
```

In addition, individual agents can have a specified location. For example:

```
%init: 1000 B:cytosol             # B distributed evenly among all voxels in cytosol
%init: 1000 C:membrane[2][2](s~u) # C in one voxel of the membrane only
```

(Saying that n copies of `A` are evenly distributed among a set of N voxels, means that all voxels receive the same number of `A`s, i.e. the quotient of n by N, the remainder being allocated randomly.)

When locations are specified both for agent groups and individual agents, the individual agent location takes precedence. This allows for concise definition of agent groups where all but one of the agents in the group share a location.

Voxel wildcards may also be used when specifying agent location. For example:

```
%compartment: cytosol [5][5][5]

%init: 1000 :cytosol[5][5][?] A # A distributed along a single edge of compartment
%init: 1000 :cytosol[?][?][2] B # B distributed in a plane across middle of compartment
```

1.3.4 Agent links

Agents in neighbouring voxels can be linked together provided their voxels are linked by a defined channel. This is an extension of the basic Kappa link syntax to name the channel used to link the agents. For example:

```
%compartment: membrane [5][5]
%compartment: cytosol  [5][5][5]

%channel: domainLink \
    (:membrane [x][y] -> :cytosol [x][y][0]) + (:cytosol [x][y][0] -> :membrane [x][y])

%init: 1000 A:membrane(d!1:domainLink), B(d!1)
```

The above describes a model where the species `AB` exists in two compartments, `B` in the cytosol and `A` embedded in the membrane. When specifying agent links using channels, only one end of the link needs to specify the channel. If a link does not specify the channel, it is assumed that both agents party to the link exist in the *same* voxel.

Links including channels can be created or broken in the same way as basic Kappa links in rules.

1.3.5 Species movement

Species can move along defined channels. Movement transition rules can either constrain the movement by species chosen, or by source location. Species movement is described using the `->:` operator.

```
(source=location)? '->:' channelName=id (target=location)?
|  (a=agentGroup)? '->:' channelName=id (b=agentGroup)?
```

For example:

```
%compartment: membrane [5][5]

%channel: diffusion \
    (:membrane [x][y] -> :membrane [x+1][y]) + (:membrane [x][y] -> :membrane [x - 1][y]) + \
    (:membrane [x][y] -> :membrane [x][y+1]) + (:membrane [x][y] -> :membrane [x][y - 1])

'diffusion A' A(s,t) ->:diffusion A(s,t) @ 1.0
'diffusion B' B(s,t) ->:diffusion B(s,t) @ 1.0
'diffusion AB' A(s!1,t),B(s!1,t) ->:diffusion A(s!1,t),B(s!1,t) @ 0.5

'diffusion all' ->:diffusion @ 1.0 # All species located in a single voxel will match this rule
```

To describe movement of species which span more than one voxel, one can use the multiple source channel definition given above (§1.3.2):

```
%compartment: membrane [5][5]
%compartment: cytosol  [5][5][5]

%channel: diffusion \
    (:membrane [x][y], :cytosol [u][v][0] -> :membrane [x+1][y],  :cytosol [u+1][v][0])  + \
    (:membrane [x][y], :cytosol [u][v][0] -> :membrane [x -1][y], :cytosol [u -1][v][0]) + \
    (:membrane [x][y], :cytosol [u][v][0] -> :membrane [x][y+1],  :cytosol [u][v+1][0])  + \
    (:membrane [x][y], :cytosol [u][v][0] -> :membrane [x][y -1], :cytosol [u][v -1][0])

%channel: domainLink \
    (:membrane [x][y] -> :cytosol [x][y][0]) + (:cytosol [x][y][0] -> :membrane [x][y])

'diffusion A' A_m:membrane(s,t,d!1:domainLink),A_c(d!1) ->:diffusion \
              A_m:membrane(s,t,d!1:domainLink),A_c(d!1) @ 1.0

'diffusion B' B_m:membrane(s,t,d!1:domainLink),B_c(d!1) ->:diffusion \
              B_m:membrane(s,t,d!1:domainLink),B_c(d!1) @ 1.0

'diffusion AB' A_m:membrane(s!2,t,d!1:domainLink),A_c(d!1), \
               B_m:membrane(s!2,t,d!3:domainLink),B_c(d!3) ->:diffusion \
               A_m:membrane(s!2,t,d!1:domainLink),A_c(d!1), \
               B_m:membrane(s!2,t,d!3:domainLink),B_c(d!3) @ 0.5
```

Fixed location constraints

It is necessary in some models to distinguish between species which can diffuse freely within all voxels of a compartment, and species which are fixed to a single voxel. The predefined location `:fixed`, used on the right hand side of a transition rule, allows this. For example, in the model below, agent `A()` can diffuse freely, while agent `B()` must remain static.

```
%compartment: cytosol [5][5][5]

%channel: diffusion Neighbour :cytosol -> :cytosol

'diffusion A' :cytosol A(),B() ->:diffusion :cytosol A(),B:fixed() @ 1.0
```

1.3.6 Instance specific reaction rates

The rate of a transition rule can depend on the composition and size of the particular species it is being applied to. This is possible for all types of transition, not just diffusion transitions.

An agent description enclosed in `||` may be used in a rate equation.

```
'|' agentGroup '|'
```

where `agentGroup` is agent definition syntax as used on the left hand side of transition rules.

When applied to particular instances of complexes, the number of matching agent structures in the complex instances chosen are counted and substituted into the rate.

For example, in the model below, diffusion rate is inversely proportional to the number of `A()` in the chosen complex

```
%compartment: array [10]
%channel: diffusion :array[x] -> :array[x+1]

%agent: A(x,y)

%init: 100 :array[0] A(x,y!1), A(x!1,y!2), A(x!2,y!3), A(x!3,y)
%init: 100 :array[0] A(x,y!1), A(x!1,y)
%init: 100 :array[0] A(x,y)

'diffusion A' A(x) ->:diffusion A(x) @ 1 / |A()|
```

In the second example, the diffusion rate depends on the agent types within individual complex instances

```
%compartment: array [10]
%channel: diffusion :array[x] -> :array[x+1]

%agent: A(x,y)
%agent: B(x,y)

%init: 100 :array[0] A(x,y!1), A(x!1,y!2), A(x!2,y!3), A(x!3,y)
%init: 100 :array[0] A(x,y!1), A(x!1,y!2), B(x!2,y!3), B(x!3,y)
%init: 100 :array[0] B(x,y!1), B(x!1,y!2), B(x!2,y!3), B(x!3,y)
%init: 100 :array[0] B(x,y!1), B(x!1,y!2), A(x!2,y!3), A(x!3,y)

'diffusion AX' A(x) ->:diffusion A(x) @ 1 / (|A()| + 10 * |B()|)
'diffusion BX' B(x) ->:diffusion B(x) @ 1 / (|A()| + 10 * |B()|)
```

In the final example, the diffusion rate depends on the states of the agents within individual complex instances

```
%compartment: array [10]
%channel: diffusion :array[x] -> :array[x+1]

%agent: A(x,y,s~y~n)

%init: 100 :array[0] A(s~y,x,y!1), A(s~y,x!1,y!2), A(s~y,x!2,y!3), A(s~y,x!3,y)
%init: 100 :array[0] A(s~y,x,y!1), A(s~y,x!1,y!2), A(s~n,x!2,y!3), A(s~n,x!3,y)
%init: 100 :array[0] A(s~n,x,y!1), A(s~n,x!1,y!2), A(s~n,x!2,y!3), A(s~n,x!3,y)
%init: 100 :array[0] A(s~n,x,y!1), A(s~n,x!1,y!2), A(s~y,x!2,y!3), A(s~y,x!3,y)

'diffusion A' A(x) ->:diffusion A(x) @ 1 / (|A(s~y)| + 10 * |A(s~n)|)
```

The `||` clause allows any agent declaration that could appear on the left hand side of a transition rule, including agents, complexes, agent state and agent location.

1.3.7 Voxel breakdown of observables

Observables using the constructs `%obs:` and `%var:` can be used to get the total amount of species per compartment. For example:

```
%obs: 'A in cytosol' :cytosol A()
%var: 'A in cytosol' :cytosol A()
```

However, for some simulations, recording the value of observables in each voxel of a compartment is useful. In these cases, using the keyword `voxel` will allow recording of observables per voxel. This works only on observable or variable declarations which specify a compartment as the location of the declaration. For example:

```
%obs: voxel 'A in cytosol' :cytosol A()
%var: voxel 'A in cytosol' :cytosol A()
```

The above each declare variables which record for each voxel within the compartment `cytosol`.

Warning - as all voxels within the compartment are recorded for each observable using the `voxel` keyword at each timepoint, the output data file will rapidly become very large.

For example models demonstrating the use of the language extensions, refer to Appendix B.

Chapter 2

Spatial Kappa simulator User Guide

2.1 Obtaining the simulator

The simulator is available from GitHub as the source Eclipse project, or as a single executable jar file. Both are available at https://github.com/lptolik/SpatialKappa/.

2.2 Starting the simulator

2.2.1 Running the executable jar

The simulator can be started by running the executable jar file:
`java -jar SpatialKappa-v2.1.1.jar`

Double clicking the jar file usually works too.

2.2.2 Running from the Eclipse project

The main class of the simulator is
`org.demonsoft.spatialkappa.ui.SpatialKappaSimulator`

Running as a Java Application will bring up the simulator.

2.3 Using the simulator

The initial screen appears as figure 2.1.

The toolbar options are:

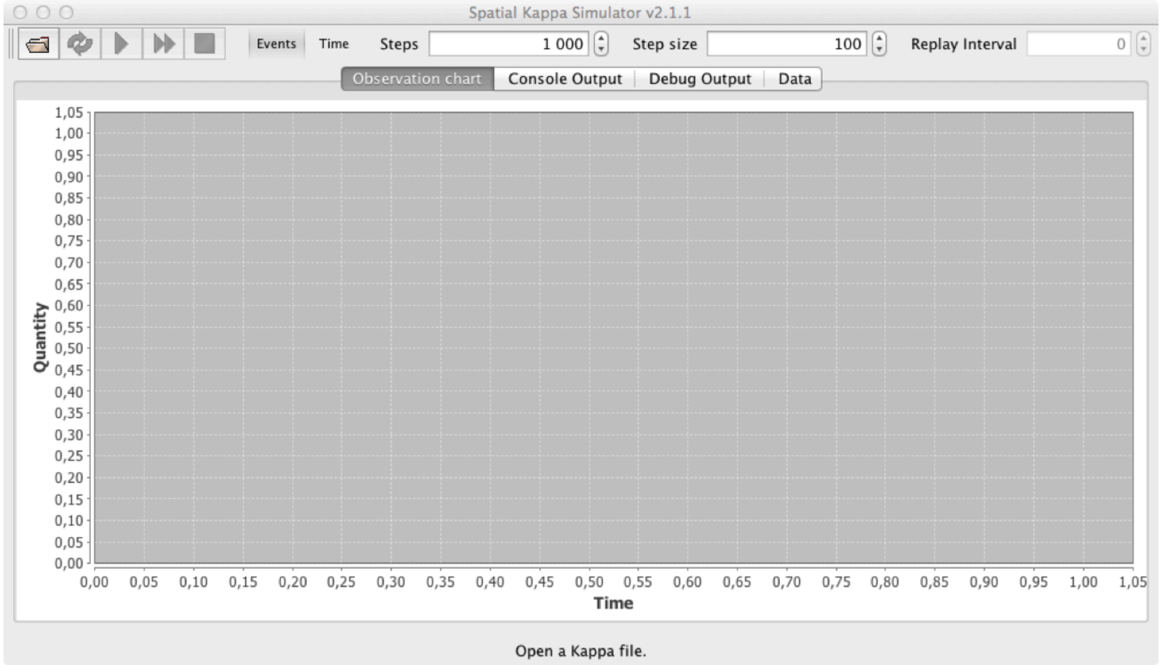

Figure 2.1: Initial view

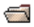	Open a Kappa or Kappa replay file
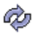	Reopen the current file
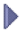	Run the current file
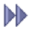	Replay the current replay file or last run
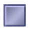	Stop the currently running simulation
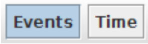	Choose event or time based simulation

2.3.1 Opening a Kappa or Kappa replay file

Select the 'Open' button on the toolbar and select the file to open. The current implementation expects Kappa source files to have the suffix `.ka` and Kappa replay files (discussed later) to have the suffix `.kareplay`. If the file is parsed successfully, a summary of the Kappa model is displayed in the 'Data' pane (see figure 2.2).

Any errors in reading the Kappa file are shown in the 'Console Output' pane.

The currently open Kappa file can be refreshed from disk by selecting the 'Reopen' button. Useful when editing the Kappa model.

2.3.2 Running a simulation

With a successfully opened Kappa model, one can run a simulation by selecting the 'Run' button. Simulation parameters can be set on the toolbar before running. There is the option to do an event or a time based simulation. For an event based simulation, the number of steps for the

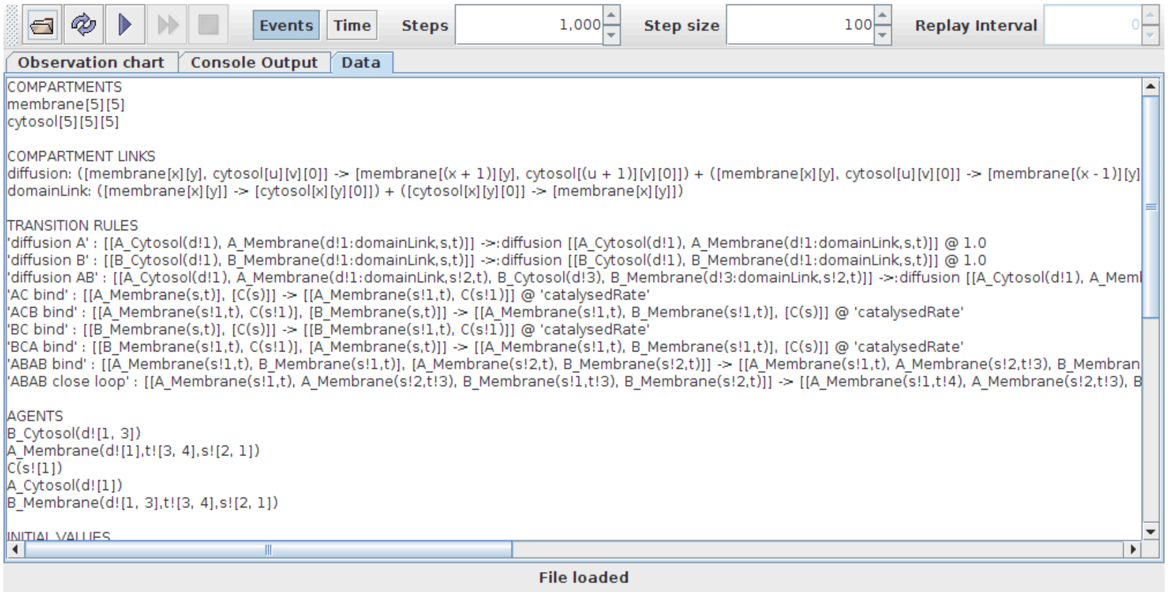

Figure 2.2: Data pane showing loaded Kappa model

simulation (i.e. data points on the time series chart), and the number of finite rate events per step can be set. Equivalent options for time based simulation can also be set.

The simulation can be halted at any point by selecting the 'Stop' button. Note that complex simulations may take some time to start up while data structures are being generated.

A replay file of the simulation is created in the same directory as the Kappa model. The file format is similar to that produced by KaSim.

2.3.3 Running a simulation replay

As the simulation runs, the state of the simulation observables are logged to disk in a replay file after every step. Once the simulation is complete, this replay file can be rerun by selecting the 'Replay' button. The 'Replay Interval' field allows a delay (in ms) to be added between each step.

The file format is similar to that produced by KaSim. Renaming KaSim data output to have the suffix `.kareplay` will allow KaSim output to also be visualised using this tool.

2.4 Time series chart

There is a simple visualisation panel in the simulator. This is dynamically updated as the simulation runs to give the user an idea of how the simulation is progressing. It is however basic in comparison to some of the commercial simulation data visualisation tools available.

The time series chart is similar to the standard Gnuplot output from KaSim. It is a line graph showing observable quantity against time for all observable definitions in the model. The excellent JFreeChart (Gilbert et al., 2010) library was used for generating the charting component. The chart has formatting and save capability, and is zoomable.

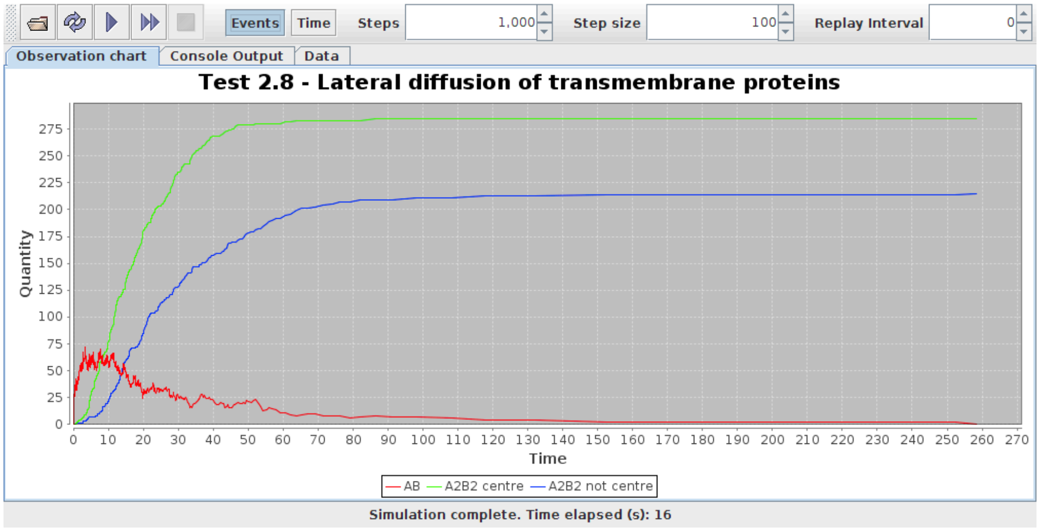

Figure 2.3: Sample time series chart output

Chapter 3

Spatial Kappa reference implementation

The algorithm implemented by Spatial Kappa simulator is similar to the Next Subvolume algorithm described in Ref. (Elf and Ehrenberg, 2004). In this chapter we briefly outline its main steps.

Apart from the differences coming from the rule-based nature of the Spatial Kappa simulator, the major difference between our algorithm and the classicalone is that there there is no distinction made between the application of a reaction event and a diffusion one. The reason for a different treatment of diffusion events in the original algorithm was the optimisation of performance. With the introduction of multi-compartmental complexes the benefit of such a different treatment has disappeared, so in Spatial Kappa all events are seen as equal in terms of the underpinning Gillespie algorithm.

3.1 Initialization

The Kappa grammar and its spatial extension are defined within the ANTLR 3.0 parser generation framework (see also Appendix A). This allows one to perform a number of initialization steps during the parsing phase, but most of the initialization rests on the simulator itself.

The rule-based nature of the framework allows to avoid some steps of initialization, for example, there is no need to build a connectivity matrix between voxels. The channel definitions play the role of rules defining diffusion reactions, in the same way as transform rules replace reaction definitions.

The first main task of the initialization is the *distribution* of agents and complexes between voxels. There are two options to do this: the model may define the exact number of agents and complexes in a particular voxel, or the total amount of agents in a compartment. In the latter case, the simulator will distribute agents homogeneously among all the compartment voxels.

The second important initialization step is to record the possible applications of reaction and diffusion rules based on the initial state. In this step one needs to calculate all possible mappings between rule left-hand-sides and the system state (a.k.a., all matchings) and their activities. The activity of the rule is the sum of activities of each of its mappings. (Rules with no mappings have zero activity.)

3.2 Main simulation loop

The main simulation loop of the Spatial Kappa simulator is the same as in Next Subvolume algorithm with modifications due to rule-based nature of the model. There are two types of model declarations that need to be processed *before* ordinary rules: perturbations and infinite rate rules.

A *perturbation* is a standard Kappa language construct that allows a model to change its state when special firing conditions are met. For example, adding a drug to the system at a particular time point, or opening ion channels when a transmembrane potential reaches some value. In the Spatial Kappa simulator perturbations are checked first in the main simulation loop and, if firing conditions are met, their modifications are applied.

It is possible to set an *infinite* rate constant for a rule. For example, to compare a Spatial Kappa model with an ordinary Kappa model, one can assign an infinite rate to well-chosen temporary diffusion rules (via perturbations) to 'stir' the model. In addition, rules which have rate constants specified by a function could receive an infinite rate depending on the system state. These require special treatment as their applications do not increment the system time. In the simulator, infinite rate rules are applied to the system after perturbation check until either there are no such rules left (or the cumulated number of applications exceeds a statically defined parameter - conventionally 1000 in the current implementation).

3.2.1 Finite rate rules

After exhausting infinite rate rules, the simulator picks one of the remaining rules and a mapping thereof and applies it to the system. The process of selecting the rule and its mapping is the same as in the Next Subvolume method: the simulator picks rule r with a probability proportional to r's activity in the current state, and selects uniformly at random a mapping of r. The rule application changes the state of the system according to r's right-hand-side, and time progresses by the same increment as in the Next Subvolume method. One then recalculates the new rule mappings and activities locally as in Ref. (Danos et al., 2007b).

Appendix A

Spatial Kappa Grammar

The following is a cut down version of the Antlr grammar used in the Kappa simulator. The syntax has been trimmed for readability, as the original Antlr grammar has artificial constructs for dealing with left recursion, etc. It is read basically as BNF notation with assignments (`variable=bnfConstruct`). The existing basic Kappa grammar is shown in `black`, with the spatial constructs shown as `blue`.

```
prog :
  (line)*

line :
  agentDecl NEWLINE!
  | compartmentDecl NEWLINE!
  | channelDecl NEWLINE!
  | ruleDecl NEWLINE!
  | initDecl NEWLINE!
  | plotDecl NEWLINE!
  | obsDecl NEWLINE!
  | varDecl NEWLINE!
  | modDecl NEWLINE!
  | COMMENT!
  | NEWLINE!

ruleDecl :
  label? transition rate

transition :
  (source=location)? CHANNEL_TRANSITION channelName=id (target=location)?
  | (a=agentGroup)? CHANNEL_TRANSITION channelName=id (b=agentGroup)?
  | (a=agentGroup)? FORWARD_TRANSITION (b=agentGroup)?

agentGroup :
  '(' agentGroup ')'
  | location? agent (',' agent)*

agent :
  id (location)? '(' (agentInterface (',' agentInterface)*)? ')'

agentInterface :
  id state? link?

state :
```

```
  '~' stateId

link :
  '!' INT ('.' channelName=id)?
  | '!' '_' ('.' channelName=id)?
  | '?'

rate :
  '@' varAlgebraExpr

initDecl :
  '%init:' (INT | label) agentGroup

agentDecl :
  '%agent:' agentName=id '(' (agentDeclInterface (',' agentDeclInterface)*)? ')'

agentDeclInterface :
  id state*

compartmentDecl :
  '%compartment:' name=id (type=id)? ('[' INT ']')*

channelDecl :
  '%channel:' linkName=id channel
  | '%channel:' linkName=id '(' channel ')' ('+' '(' channel ')')*

channel :
  (type=id)? source=locations FORWARD_TRANSITION target=locations

locations :
  location (',' location)*

location :
  ':' 'fixed'
  | ':' sourceCompartment=id compartmentIndexExpr*

compartmentIndexExpr :
  '[' '?' ']'
  | '[' cellIndexExpr ']'

plotDecl :
  '%plot:' label

obsDecl :
  '%obs:' label varAlgebraExpr
  | '%obs:' voxel? label? agentGroup

varDecl :
  '%var:' label varAlgebraExpr
  | '%var:' voxel? label agentGroup

varAlgebraExpr :
  a=varAlgebraMultExpr (op=operator_add b=varAlgebraMultExpr )*

varAlgebraMultExpr :
  a=varAlgebraExpExpr (op=operator_mult b=varAlgebraExpExpr )*

varAlgebraExpExpr :
```

```
  a=varAlgebraAtom ('^' b=varAlgebraExpExpr )*

varAlgebraAtom :
  '(' varAlgebraExpr ')'
  | number
  | label
  | '[' 'inf' ']'
  | '[' 'pi' ']'
  | '[' 'Tsim' ']'
  | '[' 'Tmax' ']'
  | '[' 'Emax' ']'
  | '[' 'T' ']'
  | '[' 'E' ']'
  | operator_unary varAlgebraAtom
  | '|' agentGroup '|'

modDecl :
  '%mod:' 'repeat' perturbationExpression 'until' booleanExpression
  | '%mod:' perturbationExpression

perturbationExpression
  '(' perturbationExpression ')'
  | booleanExpression 'do' effects

booleanExpression :
  a=booleanAtom (op=booleanOperator b=booleanAtom )*

booleanOperator :
  '&&' | '||'

relationalOperator :
  '<' | '>' | '=' | '<>'

booleanAtom :
  '(' booleanExpression ')'
  | '[' 'true' ']'
  | '[' 'false' ']'
  | '[' 'not' ']' booleanAtom
  | a=varAlgebraExpr relationalOperator b=varAlgebraExpr

effects
  '(' effects ')'
  | effect (';' effect)*

effect :
  '$SNAPSHOT'
  | '$STOP'
  | '$ADD' varAlgebraExpr agentGroup
  | '$DEL' varAlgebraExpr agentGroup
  | '$UPDATE' label varAlgebraExpr

cellIndexExpr :
  a=cellIndexAtom operator_cell_index b=cellIndexAtom
  | a=cellIndexAtom

cellIndexAtom :
  '(' cellIndexExpr ')'
  | INT
  | id
```

```
id :
  ( 'a'..'z' | 'A'..'Z' ) ( ALPHANUMERIC | '_' | '-' | '+' )*

stateId :
  ALPHANUMERIC

label :
  LABEL

number :
  ( INT | FLOAT )

operator_cell_index :
  ( '+' | '*' | '-' | '/' | '%' | '^' )

operator_unary :
  '[' 'log' ']'
  | '[' 'sin' ']'
  | '[' 'cos' ']'
  | '[' 'tan' ']'
  | '[' 'sqrt' ']'
  | '[' 'exp' ']'
  | '[' 'int' ']'

operator_mult :
  '*' | '/' | '[' 'mod' ']'

operator_add :
  '+' | '-'

CHANNEL_TRANSITION :
  '->:'

FORWARD_TRANSITION :
  '->'

INT :
  NUMERIC

FLOAT :
  NUMERIC '.' NUMERIC EXPONENT?
  | '.' NUMERIC EXPONENT?
  | NUMERIC EXPONENT

ALPHANUMERIC :
  ( NUMERIC | 'a'..'z' | 'A'..'Z' )+

NUMERIC :
  ('0'..'9')+

EXPONENT :
  ('e'|'E') ('+'|'-')? NUMERIC

LABEL :
  '\'' .* '\''

COMMENT :
  '#' ~( '\n' | '\r' )*

NEWLINE :
```

```
  '\r'? '\n' | '\r'

WS :
  ( ' ' | '\t' | '\\' NEWLINE )+
```

Appendix B

Spatial Kappa Examples

B.1 Spatial Kappa patterns

The following are generic shapes, with their equivalent Spatial Kappa representations. These are intended to be copied during model development. Explicitly specified models are shown first to demonstrate the syntax, then more concise versions are shown where possible.

B.1.1 1 dimensional patterns

Linear array

```
%compartment: array [n] # Replace n with length of array
%channel: intra-array (:array [x] -> :array [x +1]) + (:array [x] -> :array [x -1])
```

Circle

```
%compartment: circle [n] # Replace n with number of cells in circle
%channel: intra-circle \
    (:circle [x] -> :circle [x +1]) + (:circle [x] -> :circle [x -1]) + \
    (:circle [n -1] -> :circle [0]) + (:circle [0] -> :circle [n -1]) # Replace n-1 as above
```

B.1.2 2 dimensional surfaces

Rectangular mesh

There are 2 variants here, 4-way linked and 8-way linked.

```
%compartment: mesh [n][m] # Replace n and m with dimensions of mesh

# 4-way diffusion
%channel: intra-mesh \
    (:mesh [x][y] -> :mesh [x +1][y]) + (:mesh [x][y] -> :mesh [x -1][y]) + \
    (:mesh [x][y] -> :mesh [x][y +1]) + (:mesh [x][y] -> :mesh [x][y -1])

# or 8-way diffusion
%channel: intra-mesh \
    (:mesh [x][y] -> :mesh [x +1][y]) + (:mesh [x][y] -> :mesh [x -1][y]) + \
    (:mesh [x][y] -> :mesh [x][y +1]) + (:mesh [x][y] -> :mesh [x][y -1]) + \
    (:mesh [x][y] -> :mesh [x +1][y +1]) + (:mesh [x][y] -> :mesh [x -1][y -1]) + \
    (:mesh [x][y] -> :mesh [x +1][y -1]) + (:mesh [x][y] -> :mesh [x -1][y +1])
```

These can also be specified using channel types as follows

```
%compartment: mesh [n][m] # Replace n and m with dimensions of mesh

# 4-way diffusion
%channel: intra-mesh EdgeNeighbour :mesh -> :mesh

# or 8-way diffusion
%channel: intra-mesh Neighbour :mesh -> :mesh
```

Hexagonal mesh

Again, 2 variants depending on what overall shape is required. The first form has a simpler representation of intra-compartment links, but the overall structure is rhomboid, whereas the second produces an overall rectangular shape at the expense of more complicated link statements.

The second variant demonstrates handling of alternate odd-even linkage depending on the column of the structure.

```
%compartment: mesh [n][m] # Replace n and m with dimensions of mesh

# Variant 1 - rhomboid mesh
%channel: intra-mesh \
    (:mesh [x][y] -> :mesh [x][y +1]) + (:mesh [x][y] -> :mesh [x][y -1]) + \
    (:mesh [x][y] -> :mesh [x +1][y]) + (:mesh [x][y] -> :mesh [x -1][y]) + \
    (:mesh [x][y] -> :mesh [x +1][y +1]) + (:mesh [x][y] -> :mesh [x -1][y -1])

# Variant 2 - rectangular mesh
%channel: intra-mesh \
    (:mesh [x][y] -> :mesh [x][y +1]) + (:mesh [x][y] -> :mesh [x][y -1]) + \
    (:mesh [x][y] -> :mesh [x +1][y]) + (:mesh [x][y] -> :mesh [x -1][y]) + \
    (:mesh [x][y] -> :mesh [x +1][(y +1)-(2*(x%2))]) + \
    (:mesh [x][y] -> :mesh [x -1][(y -1)+(2*((x -1)%2))])

# The above statement alternates [x +1][y +1] and [x +1][y -1] as x increases
```

Variant 2 can also be specified using channel types as follows

```
%compartment: mesh [n][m] # Replace n and m with dimensions of mesh

# Variant 2 - rectangular mesh
%channel: intra-mesh Hexagonal :mesh -> :mesh
```

Cylinder and torus

By connecting together the top and bottom edges of a mesh as described above, we get a cylinder. By also connecting together the left and right edges we get a torus.

```
%compartment: mesh [n][m] # Replace n and m with dimensions of mesh

# 4-way diffusion mesh
%channel: intra-mesh \
    (:mesh [x][y] -> :mesh [x +1][y]) + (:mesh [x][y] -> :mesh [x -1][y]) + \
    (:mesh [x][y] -> :mesh [x][y +1]) + (:mesh [x][y] -> :mesh [x][y -1])
%channel: intra-mesh mesh [x][y] <-> mesh [x+1][y]
%channel: intra-mesh mesh [x][y] <-> mesh [x][y+1]

# cylinder
%channel: intra-mesh \
    (:mesh [x][y] -> :mesh [x +1][y]) + (:mesh [x][y] -> :mesh [x -1][y]) + \
```

```
    (:mesh [x][y] -> :mesh [x][y +1]) + (:mesh [x][y] -> :mesh [x][y -1]) + \
    (:mesh [x][y] -> :mesh [x][m -1]) + (:mesh [x][y] -> :mesh [x +1][0])
# Replace m-1 as above

# torus
%channel: intra-mesh \
    (:mesh [x][y] -> :mesh [x +1][y]) + (:mesh [x][y] -> :mesh [x -1][y]) + \
    (:mesh [x][y] -> :mesh [x][y +1]) + (:mesh [x][y] -> :mesh [x][y -1]) + \
    (:mesh [x][m -1] -> :mesh [x][0]) + (:mesh [x][0] -> :mesh [x][m -1]) + \
    (:mesh [n -1][y] -> :mesh [0][y]) + (:mesh [0][y] -> :mesh [n -1][y])
# Replace n-1 as above
```

The predefined compartment `OpenCylinder` allows creation of a cylinder with closed ends

```
%compartment: openCylinder  OpenCylinder  [10][8] [2]    # [diameter][length] [thickness]
```

B.2 Sample Spatial Kappa models

Here are two simple spatial kappa models which demonstrate the use of some of the language features.

B.2.1 2D diffusion model

This model shows simple diffusion from a point for three distinct species.

```
%agent: A()
%agent: B()
%agent: C()

%compartment: cytosol [30][30]

# 6-way diffusion
%channel: hexagonal \
    (:cytosol [x][y] -> :cytosol [x][y +1]) + (:cytosol [x][y] -> :cytosol [x][y -1]) + \
    (:cytosol [x][y] -> :cytosol [x +1][y]) + (:cytosol [x][y] -> :cytosol [x -1][y]) + \
    (:cytosol [x][y] -> :cytosol [x +1][(y +1)-(2*(x%2))]) + \
    (:cytosol [x][y] -> :cytosol [x -1][(y -1)+(2*((x -1)%2))])

'diffusion' ->:hexagonal @ 0.4

%init: 10000 :cytosol[10][10] A()
%init: 10000 :cytosol[10][14] B()
%init: 10000 :cytosol[14][14] C()

%obs: 'Red' A()
%obs: 'Green' B()
%obs: 'Blue' C()
```

For a more concise version, replace the explicit description of the `hexagonal` channel with:

```
# 6-way diffusion - concise version
%channel: hexagonal Hexagonal :cytosol -> :cytosol
```

B.2.2 Bi-trivalent binding model

A spatial variant of the bi-trivalent binding test model (Yang et al., 2008). Free agents can diffuse through a 2D hexagonal lattice, but bound ones remain fixed within their voxel.

```
%agent: A(a,b,bindings~0~1~2)
%agent: B(a,b,c)

%compartment: cytosol [20][20]

# 6-way diffusion
%channel: hexagonal \
    (:cytosol [x][y] -> :cytosol [x][y +1]) + (:cytosol [x][y] -> :cytosol [x][y -1]) + \
    (:cytosol [x][y] -> :cytosol [x +1][y]) + (:cytosol [x][y] -> :cytosol [x -1][y]) + \
    (:cytosol [x][y] -> :cytosol [x +1][(y +1)-(2*(x%2))]) + \
    (:cytosol [x][y] -> :cytosol [x -1][(y -1)+(2*((x -1)%2))])

'diffusion-A' A(bindings~0) ->:hexagonal A(bindings~0) @ 0.1
'diffusion-B' B(a,b,c) ->:hexagonal B(a,b,c) @ 1

A(a,b,bindings~0),   B(a) -> A(a!1,b,bindings~1),  B(a!1) @ 1
A(a,b,bindings~0),   B(b) -> A(a!1,b,bindings~1),  B(b!1) @ 1
```

```
A(a,b,bindings~0),   B(c) -> A(a!1,b,bindings~1),  B(c!1) @ 1
A(a,b,bindings~0),   B(a) -> A(a,b!1,bindings~1),  B(a!1) @ 1
A(a,b,bindings~0),   B(b) -> A(a,b!1,bindings~1),  B(b!1) @ 1
A(a,b,bindings~0),   B(c) -> A(a,b!1,bindings~1),  B(c!1) @ 1
A(a,b!_,bindings~1), B(a) -> A(a!1,b!_,bindings~2),B(a!1) @ 1
A(a,b!_,bindings~1), B(b) -> A(a!1,b!_,bindings~2),B(b!1) @ 1
A(a,b!_,bindings~1), B(c) -> A(a!1,b!_,bindings~2),B(c!1) @ 1
A(a!_,b,bindings~1), B(a) -> A(a!_,b!1,bindings~2),B(a!1) @ 1
A(a!_,b,bindings~1), B(b) -> A(a!_,b!1,bindings~2),B(b!1) @ 1
A(a!_,b,bindings~1), B(c) -> A(a!_,b!1,bindings~2),B(c!1) @ 1

A(a!1,b,bindings~1),  B(a!1) -> A(a,b,bindings~0),   B(a) @ 0.01
A(a!1,b,bindings~1),  B(b!1) -> A(a,b,bindings~0),   B(b) @ 0.01
A(a!1,b,bindings~1),  B(c!1) -> A(a,b,bindings~0),   B(c) @ 0.01
A(a,b!1,bindings~1),  B(a!1) -> A(a,b,bindings~0),   B(a) @ 0.01
A(a,b!1,bindings~1),  B(b!1) -> A(a,b,bindings~0),   B(b) @ 0.01
A(a,b!1,bindings~1),  B(c!1) -> A(a,b,bindings~0),   B(c) @ 0.01
A(a!1,b!_,bindings~2),B(a!1) -> A(a,b!_,bindings~1), B(a) @ 0.01
A(a!1,b!_,bindings~2),B(b!1) -> A(a,b!_,bindings~1), B(b) @ 0.01
A(a!1,b!_,bindings~2),B(c!1) -> A(a,b!_,bindings~1), B(c) @ 0.01
A(a!_,b!1,bindings~2),B(a!1) -> A(a!_,b,bindings~1), B(a) @ 0.01
A(a!_,b!1,bindings~2),B(b!1) -> A(a!_,b,bindings~1), B(b) @ 0.01
A(a!_,b!1,bindings~2),B(c!1) -> A(a!_,b,bindings~1), B(c) @ 0.01

%init: 600 A(a,b,bindings~0)
%init: 400 B(a,b,c)

%obs: 'Red' A(bindings~2)
%obs: 'Green' A(bindings~1)
%obs: 'Blue' A(bindings~0)
```

Bibliography

Danos, V. (2009). Agile modelling of cellular signalling. *Electronic Notes in Theoretical Computer Science*, 229(4):3–10.

Danos, V., Feret, J., Fontana, W., Harmer, R., and Krivine, J. (2007a). Rule-based modelling of cellular signalling. *CONCUR 2007 - Concurrency Theory, 18th International Conference*, (4703):17–41.

Danos, V., Feret, J., Fontana, W., and Krivine, J. (2007b). Scalable simulation of cellular signaling networks. In *Programming Languages and Systems*, pages 139–157. Springer Berlin Heidelberg.

Elf, J. and Ehrenberg, M. (2004). Spontaneous separation of bi-stable biochemical systems into spatial domains of opposite phases. *Syst. Biol*, 1(2):230.

Feret, J. and Krivine, J. (2012). *KaSim3 Reference Manual*. Available at `https://github.com/jkrivine/KaSim` as part of the KaSim source distribution.

Gilbert, D. et al. (2010). Jfreechart website http://www.jfree.org/jfreechart/.

Krivine, J., Danos, V., and Benecke, A. (2009). Modelling epigenetic information maintenance: A kappa tutorial. pages 17–32.

Yang, J., Monine, M., Faeder, J., and Hlavacek, W. (2008). Kinetic Monte Carlo method for rule-based modeling of biochemical networks. *Physical Review E*, 78(3):31910.
